# Ameliorative role of naringenin in MPTP- induced Parkinsonism: Insights from *Drosophila melanogaster* experimental model combined with computational biology

**DOI:** 10.1016/j.toxrep.2025.102004

**Published:** 2025-03-20

**Authors:** Clive Okonta, Oludare Michael Ogunyemi, Babatunde Olabuntu, Amos Olalekan Abolaji

**Affiliations:** a Drosophila Laboratory. Molecular Drug Metabolism and Toxicology Unit, Department of Biochemistry, Faculty of Basic Medical Sciences, College of Medicine, University of Ibadan, Ibadan, Oyo, Nigeria; b Structural and Computational Biology Group. Nutritional and Industrial Biochemistry Research Unit, Department of Biochemistry, Faculty of Basic Medical Sciences, College of Medicine, University of Ibadan, Ibadan, Oyo, Nigeria

**Keywords:** Parkinson's disease, Toxicity, Flavonoids, *Drosophila melanogaster*, Network pharmacology, Molecular docking

## Abstract

This study probed the ameliorative effects of naringenin in a *D. melanogaster* model of 1-methyl-4-phenyl-1,2,3,6-tetrahydropyridine (MPTP)-induced parkinsonism, incorporating computational analysis. Initially, flies were treated with naringenin (100–500 µM) and MPTP (250–750 µM) for 14 days in two separate studies to determine the optimum concentrations for the treatments. Following this, optimum naringenin concentrations (100 and 300 µM) were administered to MPTP (500 µM)-exposed flies in a 4-day study. Motor function, survival rate, and neurotoxicity biomarkers were assessed alongside biological network analysis and molecular docking simulation. Results indicate that naringenin exhibits hormetic behavior, with 100–300 µM providing optimal neuroprotection. The treatments significantly improved negative geotaxis and acetylcholinesterase activity, and reduced MPTP-induced oxidative stress as indicated by reduced nitric oxide, hydrogen peroxide, and protein carbonyl levels. Furthermore, naringenin restored thiol contents, and enhanced catalase and glutathione-S-transferase activities. Network analysis helped to identify key targets, including *DRD4, DRD2, NFKB1, MAOB, MAPK14*, and *CYP2A6*, which function in dopaminergic signaling and oxido-inflammatory pathways. Molecular docking analysis revealed strong binding interactions of naringenin with DRD2, MAO, MAPK, and NF-κB protein targets, primarily through hydrogen bonding and hydrophobic interactions. Overall, these findings suggest that naringenin mitigates MPTP-induced neurotoxicity by enhancing dopaminergic neurotransmission and suppressing oxidative stress and inflammation. This study further supports the neuroprotective potential of naringenin and could be suggested as a promising nutraceutical/drug candidate for Parkinson’s disease.

## 1 Introduction

Parkinson’s disease (PD), once considered a rare neurodegenerative disorder, is now considered the fastest growing neurological disorder which poses serious health, societal and economic threats to the low- and middle-income nations [1], [2]. It resulted in 5.8 million disability-adjusted life years (DALYs) and 329,000 deaths in 2019 [3]. The number of people affected is projected to reach 12.9 million by 2040 due to aging within populations [4]. Environmental factors including pesticides, chemicals, air pollution, and increased smoking are expected to further drive the number to a higher level, as the incidence of the disease is disproportionately increasing in emerging industrialized zones and outpacing the rate of aging [5], [6]. It is predicted that as low- and middle-income nations develop and life expectancy rises, the majority of the PD burden will arise from these countries, posing serious health and economic threats in these regions [2], [4]. Indeed, PD is a multifactorial disorder where genetic predisposition, environmental triggers, and aging combine to induce the degeneration of dopaminergic neurons in the substantia nigra causing motor symptoms including tremors, rigidity, bradykinesia (slowness of movement), and postural instability and non-motor symptoms, including cognitive decline, mood disorders, and autonomic dysfunction [7], [8]. By the time motor symptoms appear, around 60–80 % of these neurons are lost [9]. Although the hallmark feature of PD is the progressive loss of dopaminergic neurons in the substantia nigra pars compacta region of the brain which is responsible for producing dopamine, other mechanisms central to the pathogenesis of PD include alpha-synuclein aggregation, mitochondrial dysfunction, oxidative stress, and neuroinflammation [10], [11]. Levodopa (L-DOPA), the current gold standard for advanced-stage symptomatic relief and other therapeutic interventions for PD are limited and do not impede disease progression. Besides, long-term administration of L-DOPA may cause levodopa-induced dyskinesias, prompting physicians to start treatment with other dopamine agonists or monoamine oxidase inhibitors, which often fail to impede disease progression. Consequently, research is ongoing to develop new therapeutic alternatives to stop or delay PD development while protecting dopaminergic nerve cells to support proper motor function.

The 1-methyl-4-phenyl-1,2,3,6-tetrahydropyridine (MPTP) is a well-known environmental neurotoxin that is widely used in scientific research to induce PD-like symptoms in animal models [12]. Its discovery and use has been instrumental to advancing our understanding of environmental factors that contribute to the pathogenesis of PD. The MPTP-induced PD models allow researchers to investigate the molecular mechanisms of dopaminergic neuron death [13], [14], [15], [16]. Furthermore, the models are used to test the efficacy of neuroprotective agents, including antioxidants, anti-inflammatory compounds, and mitochondrial stabilizers as the model replicates both motor and non-motor symptoms of PD; and thereby provides a comprehensive tool for evaluating potential PD treatments [15], [16]. Mechanistically, MPTP enters the brain and taken up by astrocytes and then metabolized by the enzyme monoamine oxidase-B (MAO-B) into its toxic metabolite [17], [18]. The MPP+ (1-methyl-4-phenylpyridinium) is then released into the extracellular space and taken up selectively by dopaminergic neurons via the dopamine transporter (DAT), which makes dopaminergic neurons especially vulnerable to MPTP toxicity [19]. Once inside the dopaminergic neurons, MPP+ is transported into mitochondria, where it inhibits complex I of the electron transport chain [18]. This inhibition disrupts oxidative phosphorylation, leading to decreased ATP production and a subsequent increase in the generation of reactive oxygen species (ROS). The oxidative stress generated by MPP+ causes mitochondrial dysfunction, ultimately leading to energy failure, loss of membrane potential, and the activation of cell death pathways in dopaminergic neurons. Dopaminergic neurons are particularly susceptible to oxidative stress due to their high metabolic activity and the presence of dopamine, which can undergo oxidation to form toxic quinones. The resulting oxidative stress further contributes to mitochondrial dysfunction and neuronal death. MPTP-induced neurodegeneration is accompanied by activation of microglia, the resident immune cells of the brain. Microglia release pro-inflammatory cytokines, such as tumor necrosis factor-alpha (TNF-α) and interleukin-1 beta (IL-1β), which exacerbate neuroinflammation and contribute to further neuronal damage [20]. This neuroinflammatory response plays a critical role in the progression of MPTP-induced neurotoxicity and mirrors the chronic inflammation observed in human PD. The *D. melanogaster* model provides a robust and versatile platform for preclinical drug screening due to its low or absent ethical limitations, genetic tractability, short life cycle, high fecundity and low cost of maintenance. The *D. melanogaster* MPTP model is a powerful tool for studying the molecular mechanisms underlying PD and for testing potential neuroprotective treatments as many of the core processes involved in PD pathogenesis, such as mitochondrial dysfunction, protein aggregation, and neurodegeneration, are well-conserved in *D. melanogaster* [16], [21]. Chronic administration of MPTP to flies has been shown to cause selective degeneration of dopaminergic neurons, neuroinflammation and locomotor abnormality, mimicking the symptoms of parkinsonism [22].

Computational biology tools and data integration techniques including algorithms, simulations, and molecular docking techniques for predicting how drugs interact with biological targets continue to evolve towards revolutionizing drug discovery. Network pharmacology focuses on understanding how drugs/toxins interact with multiple targets within biological networks, rather than the traditional single-target drug development model. This multi-dimensional approach integrates computational biology, bioinformatics, and pharmacology to study the interactions between drugs, targets, and biological systems on a network level. By focusing on multi-target interactions, system robustness, and network modularity, network pharmacology offers a more integrative and holistic approach to understanding how drugs work within the broader context of human health and disease. This shift enables the development of more effective therapies for complex diseases, reduces adverse effects, and supports the emerging field of personalized medicine [23]. Several studies have reported identification of multitargeting phytochemicals against parkinsonism through network pharmacology [24], [25], [26]. While network pharmacology is a powerful tool for identifying potential drug targets, molecular docking and other molecular modelling tools can help to clarify the interaction of the drug with these multiple targets. Molecular docking integrated with molecular dynamics simulation has been used to suggest various natural products that interacts with important targets in neurodegenerative diseases [27], [28]. Integration of network pharmacology with molecular docking ensures that drugs can modulate different nodes within a network; and thereby enables the discovery of multi-target drugs and the development of more effective therapies for complex diseases such as neurodegenerative diseases. Combining *in vivo* studies with computational modeling offers a powerful approach to understanding biological processes and developing therapeutic interventions. This integrative approach is particularly valuable in fields like neurodegenerative diseases, pharmacology, and toxicology, where both animal models and computational tools can provide complementary insights.

Dietary polyphenols are natural compounds found in food plants that have gained attention for their potential as promising therapeutic agents. These compounds, which include flavonoids, phenolic acids, lignans, and stilbenes, are abundant in fruits, vegetables, tea, wine, and cocoa. Due to their wide range of biological activities, polyphenols are being explored as drugs for various chronic diseases, particularly because of their safety and multifunctional properties [29], [30], [31]. Our previous studies have revealed the protective role of some flavonoids including of resveratrol, hesperetin and curcumin in *D. melanogaster* models [32], [33], [34]. Naringenin, a flavonoid found predominantly in citrus fruits, has demonstrated significant neuroprotective properties through its antioxidant, anti-inflammatory, and anti-apoptotic activities, as well as its ability to protect mitochondria and enhance neurogenesis and synaptic plasticity. These mechanisms collectively make naringenin a promising therapeutic agent for neurodegenerative diseases. The neuroprotective mechanism of naringenin is not fully understood in *D. melanogaster*, a versatile model for current and future preclinical studies of this compound which can further elucidate its potential as a neuroprotective agent in human health. Therefore, this study focused on exploring the beneficial effects of naringenin in *D. melanogaster* model of MPTP-induced parkinsonism combined with network pharmacology and molecular docking.

## 2 Materials and methods

### 2.1 Chemicals and *D. melanogaster* culture

All chemicals used were commercial products of analytical grade. The 1-methyl-4-phenyl-1,2,3,6-tetrahydropyridine (MPTP) at a percentage purity of 95 % and naringenin were procured from A K Scientific, 30023 Aherm Ave, Union City, CA 94587, United States of America. The 5′,5′-dithiobis(2- nitrobenzoic acid) (DTNB), and reduced glutathione (GSH) were purchased from Sigma Aldrich (St. Louis, MO, USA).

### 2.2 Culture of *D.melanogaster* culture

The *D. melanogaster* (wild type, Oregon strains) flies (1–3 days old) from the National Species Stock Centre, Bowling Green, Oklahoma, USA were used for this study. They were obtained from the Department of Biochemistry and Molecular Biology, Federal University of Santa Maria, Brazil. The flies were maintained and reared on cornmeal medium mixed with brewer's yeast (1 % w/v), agar-agar (1 % w/v), and nipagin (preservative, 0.08 % v/w) at constant temperature (23 ± 2 °C) under 12 h dark/light cycle in the Drosophila Laboratory, Department of Biochemistry, University of Ibadan, Oyo-State, Nigeria.

### 2.3 MPTP exposure and naringenin treatment

To determine the appropriate doses and duration of naringenin treatment and MPTP exposure a 14-day survival study was carried out on two separate sets of both *D. melanogaster* genders (1–3 days old) as previously described earlier [35]. Briefly, different groups containing 50 flies/vial (n = 5) were orally exposed to naringenin (100, 200, 300, 400, and 500 µM) for 14 days. Daily mortality was enumerated and used to estimate the percentage of surviving flies in each vial. Also, the oxidative stress markers and antioxidant status of the flies were determined. Similarly, flies were exposed to MPTP (250, 500, and 750 µM) for 14 days and percentage of surviving flies were estimated. Based on the data obtained, 100 and 300 µM of naringenin as well as 500 µM of MPTP were selected and exposed to flies for 4 days. Thereafter, indices of survival, neurotoxicity, inflammation, oxidative stress and antioxidant status were estimated. Locomotor performance of naringenin- and MPTP-treated flies was investigated using the negative geotaxis assay method [36] as described in a previous study [37].

### 2.4 Biochemical analyses

Following the treatments, the flies were anaesthetized using carbon (iv) oxide, weighed, and homogenized in 0.1 M phosphate buffer, pH 7.0 (ratio of 1 mg:10 mL). Centrifugation of the homogenates was performed at 4000 g for 10 mins at 4°C in a Thermo Scientific Sorval Micro 17 R refrigerated centrifuge. Supernatants were separated into labeled conical tubes, stored at −20°C and aliquots used for the determination of biochemical parameters carried out in duplicates for each of the five replicates of MPTP and naringenin concentrations. Estimation of protein was carried out using Lowry’s method [38]. Determination of Total thiol level was achieved using Ellman’s method [39]. The enzyme activity of Glutathione-S-transferase was assayed following the method of Habig and Jakoby [40]. Catalase activity was assayed according to the method described by Aebi [41]. The procedure of Ellman, et al. [42] was used for evaluating the activity of acetylcholinesterase (AChE). The H_2_O_2_ content was estimated using the method of Jiang, et al. [43]. The nitrite level in the aliquots was determined using the Griess reaction method [44].

### 2.5 Gene mining, target prediction and network construction

The SMILES notation and the 2D chemical structure of naringenin were used on the Swiss Target Prediction database (http://www.swisstargetprediction.ch) [45] and PharmMapper (http://www.lilab-ecust.cn/pharmmapper/) [46] to predict target genes. The predicted target genes were imported into the UniProt database (https://www.uniprot.org/) [47] to retrieve the standard gene names, while duplicate entries were removed. The GeneCards database (https://www.genecards.org/) [48] and Online Mendelian Inheritance in Man (OMIM, http://omim.org/) [49] were used to predict potential MPTP- induced targets. The related targets were collected using the keywords “1-methyl-4-phenyl-1, 2, 3, 6-tetrahydropyridine-induced parkinson’s disease” and “parkinsonism” along with the Homo sapiens species. In order to identify the intersection genes as potential targets, a Venn diagram was created by using Draw Venn Diagram tool (https://bioinformat ics.psb.ugent.be/webtools/Venn/) accessed on 11 September, 2024. The UniProt database was utilized to retrieve the standard gene names, and after merging the predicted targets for the three keywords from both databases, duplicate entries were removed. Protein–protein interaction (PPI) networks were constructed using the STRING (https://cn.string-db.org/cgi/input.pl) [50]. The PPI networks were imported into Cytoscape 3.10.2 [51] software to obtain the hub genes. The cytoHubba plugin [52] was utilized to compute the top 10 hub nodes using the degree (k). The Analyze Network tool was used for topological analyses of the network, including Maximal Clique Centrality (MCC) method, degree (k), betweenness centrality (BC), clustering coefficient, closeness centrality (CC), and average shortest pathway (ASPL). The overlapping genes were further explored through Gene Ontology (GO) and Kyoto Encyclopedia of Genes and Genomes (KEGG) enrichment analyses using the Shiny GO 0.77 tool (http://bioinformatics.sdstate.edu/go/ [53], with an FDR of <0.05 and p of <0.05 as cut-off values, displaying the top ten results.

### 2.6 Molecular docking

The 3-D crystallographic structures of selected key target proteins were retrieved from Protein Database (http://www.rcsb.org). These include DRD2 (PDBID: 8IRS) complexed with agonist rotigotine, MAOB (PDBID: 2V60) in complex with inhibitor 7-[(3-Chlorobenzyl)oxy]-2-Oxo-2h-Chromene-4-Carbaldehyde (safinamide), MAPK/JNK3 (PDBID: 7S1N) cocrystalized with inhibitor 4-[5-(2-chloro-6-fluoroanilino)-6-methyl-1H-pyrazolo[3,4-*b*]pyridin-1-yl]-N-(oxetan-3-yl)thiophene-2-carboxamide (DA-74674) and NF-kB (PDBID: 2O61). All the co-crystallised/reference compounds and water molecules were deleted and missing hydrogen atoms were added using MGL-AutoDock Tools (ADT, v1.5.6) as demonstrated earlier [54]. The Kollamn charges were added as the partial atomic charge [55]. The 3D structures of naringenin and reference compounds were downloaded from the PubChem compound database (www.pubchem.ncbi.nlm.nih.gov) as Structure data format (SDF) files. The SDF files of the ligands were then converted to mol2 chemical format using Open babel [56]. The Gasteiger-type polar hydrogen charges were assigned to the atoms in the structures, while the non-polar hydrogen atoms were merged with the carbons. The internal degrees of freedom and torsions were set to zero as demonstrated earlier [30]. Finally, the structure files were converted to the PDBQT format dockable in AutoDock Tools. The structures of the hub targets were retrieved from the Protein Data Bank (https://www.rcsb.org). The structures of naringenin and the native compounds were imported into AutoDock Vina incorporated in PyRx 0.8. The structures were then minimized through Open Babel using the Universal Force Field (UFF) as the energy minimization parameter and conjugate gradient descent as the optimization algorithm. The small molecule compounds were then docked against the binding/active sites of the proteins. The binding/active sites were each defined by the grid boxes covering the active site residues as demonstrated earlier [57], [58]. The docking was performed with other parameters kept as default. Subsequently, visual inspection was performed on the various docked posed using Discovery Studio Visualizer version 16.

### 2.7 Statistical analyses

Data were expressed as mean ± standard error of mean. Comparison of data was carried out by one-way ANOVA. Statistical significance was set at P < 0.05.

## 3 Results

### 3.1 Naringenin alone improves longevity and antioxidant status in *D. melanogaster*

**Fig. 1:**
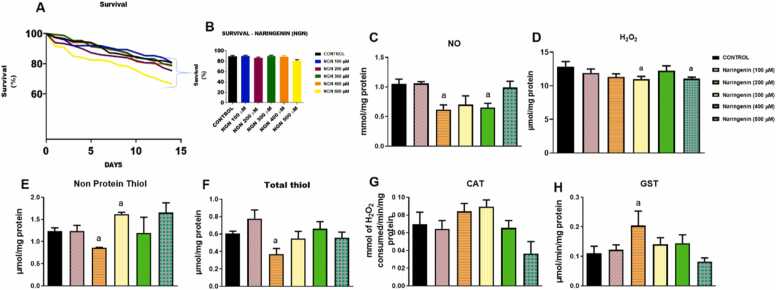
Effects of different concentrations of naringenin treatments on the survival and biochemical indices in *D. melanogaster*. (A) Survival curves of *D. melanogaster* under various concentrations of naringenin (100, 200, 300, and 500 μM) over a 14-day period. (B) Bar graph representing the percentage survival of *D. melanogaster* on day 14 across the different naringenin concentrations. (C-H) oxido-inflammation markers viz: Nitric oxide (NO), H2O2 (H_2_O_2_), Total thiol, non-protein thiol, catalase (CAT) enzyme activities and Glutathion S- transferase activity (GST). Each bar in the bar graphs represents the mean ± SEM for each group, with statistical significance marked for differences compared to the control (P < 0.05).

Fig. 1 shows the effects of naringenin supplementation on survival and oxidative/antioxidant markers in *D. melanogaster.* For the survival test, a representation of mortality over a period of 14 days in treated and untreated flies is presented in Fig. 1A. The survival rates show a downward trend over time, with some differences based on concentration (Fig. 1A). Upon a 14-day incubation of flies with normal diet, the percentage of surviving flies was observed as 89.37 % (Fig. 1B). However, this value was observed as 90.22 % (P < 0.05), 86.18 % (P < 0.05), 89.73 % (P < 0.05), 88.89 % (P < 0.05) and 80.67 % in flies fed with diets supplemented with concentrations of 100, 200, 300, 400 and 500 µM respectively indicating that, survival is quite high in the lower concentrations (100 and 300 µM) (Fig. 1B). Higher concentrations of naringenin (400 and 500 µM) seem to have a negative impact on survival compared to lower concentrations or control, indicating that, higher doses of naringenin might induce toxicity, whereas lower doses appear less harmful or neutral with respect to survival. Figures C-H show the results of the effects of naringenin on the levels of various biochemical markers related to oxidative stress and antioxidative defense. Antioxidants like SOD, CAT are elevated at lower concentrations of naringenin but may decrease at higher concentrations. Similar to the survival test, naringenin might have a hormetic effect on the biochemical indices, where lower concentrations enhance antioxidant defenses and higher concentrations may overwhelm the system and lead to oxidative damage.

### 3.2 Naringenin reduces toxicity in MPTP‑treated *D. melanogaster*

**Fig. 2:**
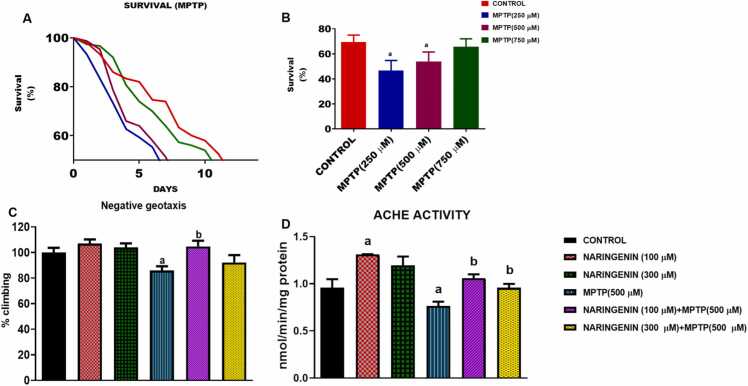
Effects of MPTP and naringenin on survival, locomotor function, and acetylcholine esterase activity in *D. melanogaster*. (A) Survival curves of *D. melanogaster* treated with different concentrations of MPTP compared to control flies. MPTP treatments lead to a significant reduction in survival over the course of 14 days, indicating its neurotoxic effects. The survival rate in MPTP-treated flies declines sharply compared to the control group. (B) The survival rate of the flies on the 12th day. (C) Bar graph representing the beneficial effects of 300 and 500 μM naringenin on the negative geotaxis behavior of MPTP-treated flies. Naringenin treatment shows a dose-dependent improvement in climbing ability, with higher concentrations restoring motor function more effectively compared to the MPTP-only group. (D) Acetylcholinesterase (AChE) activity levels in MPTP-treated flies with and without naringenin. MPTP causes a significant reduction in AChE activity, indicating its neurodegenerative impact. Naringenin supplementation shows a protective effect by increasing AChE activity, particularly at higher concentrations, mitigating the neurotoxic effects of MPTP. Each bar represents the mean ± SEM for each group, with statistical significance denoted for comparisons with the control and MPTP-treated groups (P < 0.05).

Fig. 2A depicts the effects of different concentrations of MPTP (250 µM, 500 mM, 750 mM) on *D. melanogaster* survival over time, compared to a control group. The data show that higher concentrations of MPTP result in more rapid declines in survival. Specifically, after 14 days incubation period, survival rates decreased to 46.53 %, 53.90 % and 65.78 % in flies exposed to diets supplemented with 250 µM, 500 µM, and 750 µM of MPTP respectively (Fig. 2B). Fig. 2 also shows the results of the effects of different concentrations of naringenin combined with MPTP treatment on neurotoxicity. Assessment of the motor function using the flies' ability to climb (negative geotaxis) revealed that, MPTP impairs climbing ability, consistent with neurodegenerative damage (Fig. 2C). Naringenin helped restore motor function impaired by MPTP, with the most effective improvement seen at moderate doses. Higher doses do not seem to provide additional benefits. The basal AChE enzyme activity in flies fed with a normal diet was 0.96 ± 0.16 nmol/min/mg protein. The addition of MPTP to the diet reduced this rate by 20.8 % to 0.76 ± 0.09 (P < 0.05) (Fig. 2D). In the presence of naringenin alone, AChE activities was significantly increased to 1.31 ± 0.004 (P < 0.05) at concentrations of 100 µM. However, the flavonoid restored MPTP-induced inhibition of AChE to 1.06 ± 0.08 and 0.96 ± 0.08 (P < 0.05) at concentrations of 100 µM and 300 µM respectively (Fig. 2D). The results show marked improvement compared to the MPTP-only group, while suggesting again that high naringenin doses may not be as effective or might even reduce AChE activity. Naringenin appears to counteract the MPTP-induced reduction in AChE activity, with moderate doses having the best effect. Higher doses are less beneficial or potentially harmful.

### 3.3 Role of naringenin in oxido-inflammatory modulation in MPTP‑induced toxicity

**Fig. 3:**
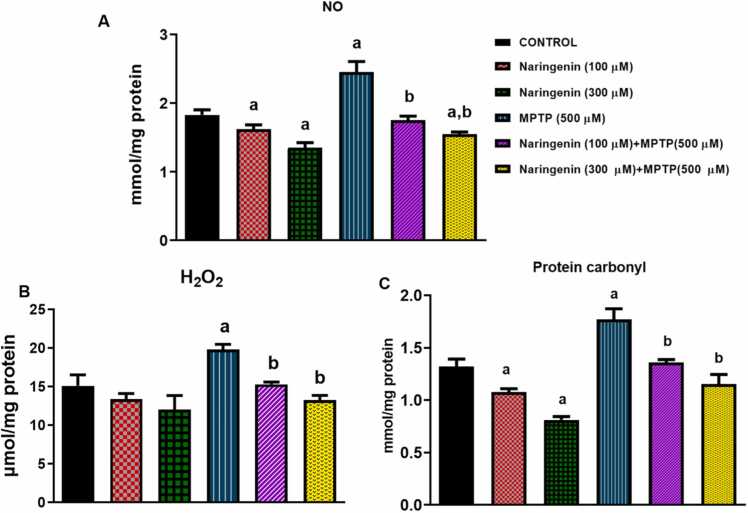
Beneficial effects of naringenin on oxidative stress markers in *D. melanogaster* exposed to MPTP-induced toxicity. (A) The levels of nitric oxide (NO) in control, MPTP-treated, and naringenin-treated flies. MPTP exposure significantly elevates NO levels compared to the control group. Naringenin treatment at different concentrations shows a dose-dependent reduction in NO levels, indicating a protective effect against MPTP-induced oxidative stress. (B) The levels of H₂O₂ in the various experimental groups. MPTP-treated flies exhibit a marked increase in H₂O₂ levels, reflecting oxidative damage. Naringenin supplementation significantly decreases H₂O₂ levels, with higher concentrations providing better protection, as seen by the reduced levels compared to the MPTP-only group. (C) Protein carbonyl levels in control, MPTP, and naringenin-treated flies. MPTP treatment leads to a notable increase in protein carbonylation, indicative of protein oxidative damage. Naringenin administration reduces protein carbonyl levels in a concentration-dependent manner, suggesting naringenin’s potential in mitigating MPTP-induced protein damage. Each bar represents the mean ± SEM for each group, with statistical significance marked for comparisons to the control and MPTP-treated groups (P < 0.05).

In this study, the effects of naringenin on MPTP-induced oxidative stress are evaluated, specifically focusing on three markers: NO, H_2_O_2_, and protein carbonyls in *D. melanogaster* as depicted in Fig. 3. The basal concentration of NO in flies fed with normal diet was observed as 1.83 ± 0.13 mmol/mg protein. Diet supplementation with MPTP (500 µM) significantly increased this value by 1.3-fold to 2.45 ± 0.27 mmol/mg protein (P < 0.05, Fig. 3A). Naringenin alone affected the rate of NO production in fruit flies at concentrations of 100 µM (1.62 ± 0.12 mmol/mg protein) and 300 µM (1.35 ± 0.13 mmol/mg protein) (P < 0.05). Also, naringenin inhibited the elevation of NO production caused by MPTP (500 µM) (Fig. 3A). The basal concentration of H_2_O_2_ flies fed with unsupplemented diets was observed as 15.11 ± 2.46 µmol/mg protein. The addition of MPTP (500 µM) to the diet increased this value by 1.3-fold to 19.80 ± 1.21 µmol/mg protein (P < 0.05) (Fig. 3B). No significant change was observed in the levels of H_2_O_2_ in flies treated with naringenin alone. However, naringenin at concentrations of 100 µM (15.30 ± 0.61 µmol/mg protein) and 300 µM (13.28 ± 1.16 µmol/mg protein) inhibited the elevation of H_2_O_2_ in flies receiving both the flavonoid and MPTP diet supplementation (Fig. 3B). For protein carbonyl production, basal levels in flies fed with normal diet was observed as 1.32 ± 0.14 mmol/mg protein. Diet supplementation with MPTP (500 µM) significantly increased this value by 1.3-fold to 1.77 ± 0.20 mmol/mg protein (P < 0.05, Fig. 3C). Naringenin alone affected the rate of protein carbonyl production in fruit flies at concentrations of 100 µM (1.08 ± 0.06 mmol/mg protein) and 300 µM (0.81 ± 0.06 mmol/mg protein) (P < 0.05). Also, naringenin restored the elevated protein carbonyl production caused by MPTP (500 µM) by 23.7 %. to 1.36 ± 0.05 mmol/mg protein (P < 0.05) and 1.15 ± 0.18 mmol/mg protein (P < 0.05) at concentrations of 100 µM and 300 µM respectively (Fig. 3C).

**Fig. 4:**
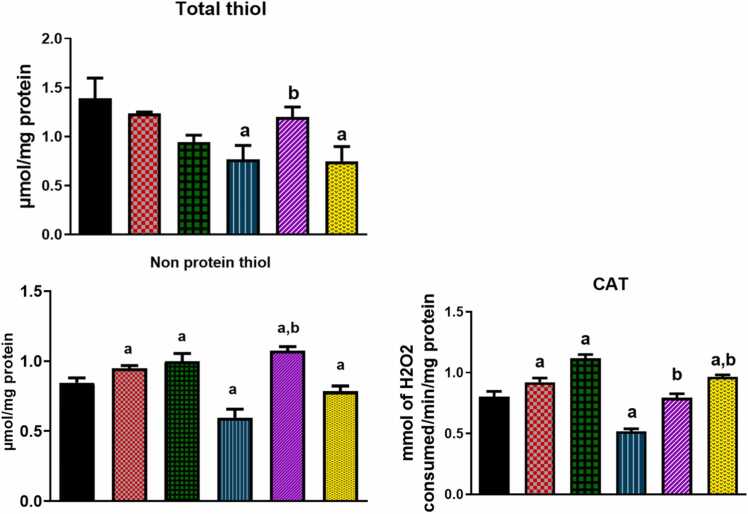
The MPTP and Naringenin on antioxidant status in *D. melanogaster* exposed to MPTP-induced toxicity (A) The level of Total protein thiol oxide in control, MPTP-treated, and naringenin-treated flies. (B) Non-protein thiol level (C) Catalase activity in control, MPTP-treated, and naringenin-treated flies and (D) Glutathione-S-transferase enzyme activity. Values are expressed as mean ± standard deviation (n = 5). Each bar represents the mean ± SEM for each group, with statistical significance marked for comparisons to the control and MPTP-treated groups (P < 0.05).

Total thiol, non-protein thiol, as well as catalase (CAT) and glutathione-S-transferase (GST) enzyme activities were used as markers of anti-oxidant status in this study. Fig. 4 shows that, naringenin ameliorates the MPTP-induced decrease levels of total thiol and non-protein thiol as well as CAT and GST enzyme activities in *D. melanogaster*. The basal total thiol levels in flies fed with unsupplemented diets was observed as 1.39±0.36 µmol/mg protein. The addition of MPTP (500 µM) to the diet depleted total thiol concentration by 44.6 % to 0.77±0.34 µmol/mg protein (P < 0.05) (Fig. 4 A). No significant depletion of total thiol was observed in flies treated with naringenin only. However, naringenin at 100 µM inhibited the reduction in total thiol levels in flies receiving both the flavonoid and MPTP diet supplementation (Fig. 4A). With respect to non-protein thiol levels, basal concentration in flies fed with normal diet was observed as 0.85±0.07 µmol/mg protein. Diet supplementation with MPTP (500 µM) decreased this value by 29.4 % to 0.60±0.11 µmol/mg protein (P < 0.05, Fig. 4B). Naringenin at concentrations 100 µM and 300 µM were found to significantly increase the levels of non-protein thiol when compared with unsupplemented diet. However, naringenin restored the depletion of non-protein thiol caused by MPTP to 1.07±0.05 µmol/mg protein and 0.78±0.08 µmol/mg protein at concentrations of 100 µM and 300 µM respectively (Fig. 4B). For the GST activities, basal enzyme activities in flies fed with normal diet was 0.57±0.05 µmol/min/mgprotein. The addition of MPTP to the diet reduced this rate by 42.1 % to 0.33±0.05 µmol/min/mgprotein (P < 0.05) (Fig. 4 C). In the presence of naringenin alone, GST activities were significantly increased to 0.70±0.08 µmol/min/mgprotein and 0.84±0.04 µmol/min/mgprotein (P < 0.05) at concentrations of 100 µM and 300 µM respectively. Moreover, the flavonoid restored GST inhibition observed in MPTP treated flies to 0.57±0.06 µmol/min/mgprotein and 0.61±0.08 µmol/min/mgprotein (P < 0.05) at concentrations of 100 µM and 300 µM respectively (Fig. 4 C). Similarly, the basal CAT enzyme activities in flies fed with normal diet was 0.80±0.09 mmol of H_2_O_2_ consumed/min/mg protein. The addition of MPTP to the diet reduced this rate by 35 % to 0.52±0.04 mmol of H_2_O_2_ consumed/min/mg protein (P < 0.05) (Fig. 4D). In the presence of naringenin alone, catalase activities were significantly increased to 0.92±0.06 mmol of H_2_O_2_ consumed/min/mg protein and 1.12±0.06 mmol of H_2_O_2_ consumed/min/mg protein (P < 0.05) at concentrations of 100 µM and 300 µM respectively. Moreover, the flavonoid restored catalase inhibition observed in MPTP treated flies to 0.79±0.06 mmol of H_2_O_2_ consumed/min/mg protein and 0.97±0.03 mmol of H_2_O_2_ consumed/min/mg protein (P < 0.05) at concentrations of 100 µM and 300 µM respectively (Fig. 4D).

### 3.4 Targets network and protein-protein interactions

**Fig. 5:**
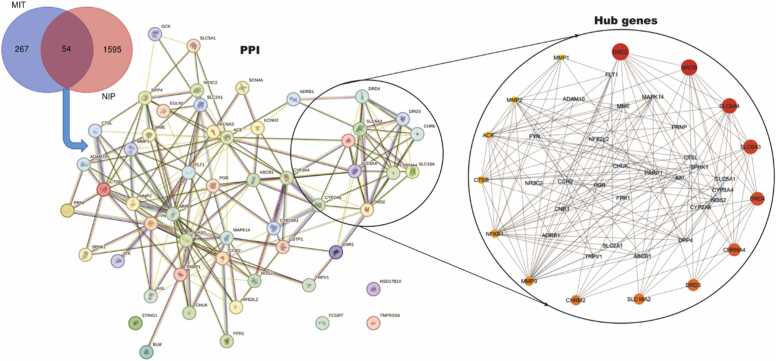
Protein-Protein Interaction (PPI) Network of Genes and Identification of Hub Genes in of MPTP-induced toxicity (MIT) and Naringenin-induced protection (NIP) in parkinsonism model. The Venn diagram illustrate the common genes. The interaction network (middle section) visualizes the relationships among these proteins, with edges representing interactions. The inset on the right zooms into the hub genes, which are identified based on their high degree of connectivity in the network.

Hub genes that may drive the ameliorative potential of naringenin in MPTP-induced parkinsonism were identified through gene mining, target prediction and network construction as depicted in Fig. 5. The Venn diagram shows targets relevant to MPTP-induced toxicity (MIT) and the target genes in naringenin-induced protection (NIP) in Parkinsonism. The MIT targets contain 267 genes that are specifically targeted in MPTP-induced Parkinsonism but are not targeted by naringenin. The NIP targets includes 1595 genes targeted by naringenin, which are not directly involved in the MPTP-induced Parkinsonism model. The overlap between the two target sets contains 54 genes that are common to both MPTP-induced Parkinsonism and naringenin amelioration. Evaluation of the Protein-Protein Interaction (PPI) network of the common target genes revealed 54 nodes and 186 edges, where nodes represent proteins and edges represent interactions between them. The interconnected nature of the network suggests a complex web of interactions among the common target genes. The network shows a variety of interaction types, indicated by the different colors of the edges, which represent various forms of interaction data, such as physical binding, co-expression, or pathways. Specific proteins are central and highly connected within the network, acting as hubs. These hub proteins may play critical roles in the overall function and stability of the network. Proteins including DRD4, DRD2, NFKB1, MAOB, MAPK14, and CYP2A6 are prominently connected, suggesting that they may be central to the shared pathways between Parkinsonism and naringenin's ameliorative effects.

Identification, visualization and ranking of the hub genes based on Maximal Clique Centrality (MCC) algorithm using cytoHubba, with Maximal Clique Centrality (MCC) algorithm revealed the hub targets as shown in Fig. 5. The hub genes are represented as nodes with different sizes and colors, indicating their importance in the network. In addition to MCC, more topological metrics were computed using various algorithms as presented in Table 1.

**Table 1:** Topological metrics of the extracted hub genes.

Nodes	Protein names	MCC	k	CC	EPC	BottleNeck	EcCentricity	BC
DRD2/D2R	Dopamine Receptor D2	2185	12	27.25	22.30	4	0.309	141
MAOB	Monoamine Oxidase B	2184	11	26.91	20.90	7	0.231	105
SLC6A4	Serotonin Transporter	2180	12	28.08	20.92	1	0.309	184
SLC6A3/DAT	Dopamine Transporter	2168	10	23.83	18.77	1	0.231	26
DRD4/D4R	Dopamine Receptor D4	2162	9	22.17	13.53	1	0.231	9
CHRNA4	Cholinergic Receptor Nicotinic Alpha 4 Subunit	1446	8	22.42	18.06	1	0.231	7
DRD3/D3R	Dopamine Receptor D3	1440	7	21.50	16.55	1	0.231	0.3
SLC18A2/ VMAT2	Vesicular Monoamine Transporter 2	720	14	21.00	21.82	1	0.231	0
CHRM2	Muscarinic Acetylcholine Receptor M2	720	6	21.00	15.92	1	0.231	0
MMP9	Matrix Metallopeptidase 9	682	21	33.50	25.68	9	0.308	259
NFKB	Nuclear Factor Kappa B	554	19	32.83	25.14	2	0.308	264
CSTB	Cystatin B	388	12	27.92	22.68	3	0.231	82
ACE	Angiotensin-Converting Enzyme	383	16	19.58	24.95	10	0.231	1
MMP2	Matrix Metallopeptidase 2	306	14	29.83	24.33	1	0.308	77
MMP1	Matrix Metallopeptidase 1	246	4	25.50	19.79	2	0.308	10

NB: MCC (Maximal Clique Centrality), k (Degree Centrality), CC (Closeness Centrality), EPC (Edge Percolation Centrality), BC (Betweenness Centrality)

The results of the topology analysis of the hub genes provide valuable insights into the structure and importance of these genes within the network related to MPTP-induced toxicity and naringenin-induced amelioration. The DRD2/D2R, the topmost protein based on the MCC value (2185), indicate a very high maximal clique centrality; and thereby act as a key connector in the network. Its Degree (k) value (12) reflects its number of direct interactions with other genes. A high EPC value (22.30) of this protein suggests its significant role in maintaining network connectivity. The relatively high EcCentricity value (0.309) implies that its distance to the farthest node in the network is moderate, while its Betweenness value (141) indicates a significant control over the flow of information within the network. The MAOB ranked second after DRD2 based on the MCC value (2184). Although this value is slightly lower, the BottleNeck value (7) shows a more prominent role in controlling flow compared to DRD2. The MCC value of SLC6A4 (2180) closely follows DRD2 and MAOB, while the EcCentricity (0.309) is similar to DRD2. The MCC value of SLC6A3 (2168) is slightly lower but still significant. MMP9 exhibited the highest Closeness value (33.5). DRD4 also maintains maintaining high centrality (MCC = 2162).

### 3.5 Functional and pathway enrichment of hub targets

**Fig. 6:**
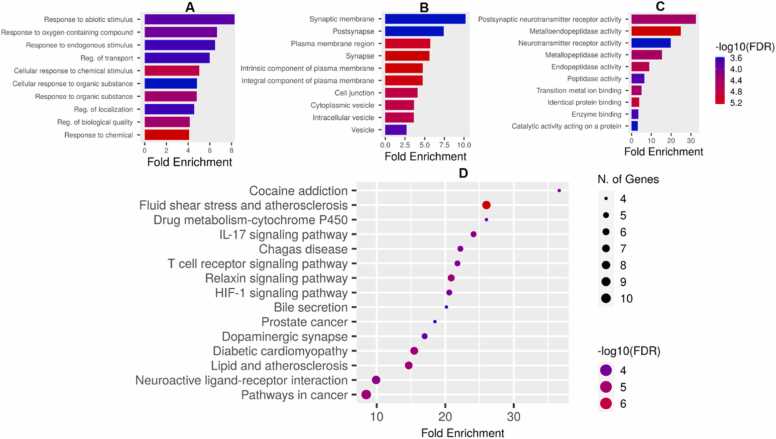
Functional Enrichment Analysis of Naringenin-Targeted Genes in PD. The figure presents the gene ontology (GO) and pathway enrichment analysis of genes targeted by naringenin in the context of PD, emphasizing their biological processes (A), cellular components (B), molecular functions (C), and KEGG pathways (D). The fold enrichment and false discovery rate (FDR) significance are color-coded, with red shades representing the highest significance in all panels.

It is important to understand the biological and biochemical relevance of the hub proteins. Therefore, the Gene Ontology (GO) enrichment and pathway analyses were performed. The Biological Process enrichment analysis shown in Fig. 6A highlights several processes that are significantly enriched among the hub genes. Response to abiotic stimulus has the highest level of enrichment. Response to Oxygen-Containing Compound is also highly enriched. High enrichment in regulation of transport suggests the involvement of hub genes in regulating the transport of molecules across cell membranes. The Cellular Component enrichment analysis of the hub genes, as shown in Fig. 6B reveals key cellular locations where these genes are most active. Among the cellular components, Synaptic Membrane has the highest enrichment, indicating a strong association of the hub genes with synaptic membranes. In a similar manner, postsynapse is highly enriched, showing the importance of the hub genes in the postsynaptic region. A general enrichment within the synapse as a whole was observed. Strong enrichment of Cytoplasmic Vesicle and Intracellular Vesicle was observed. Fig. 6C illustrates the fold enrichment of various molecular functions. The Postsynaptic Neurotransmitter Receptor Activity is the most enriched. This term involves the function of proteins that act as receptors for neurotransmitters in the postsynaptic membrane, particularly in neurotransmission. Metalloendopeptidase Activity was also highly enriched. Besides the MMPs, other proteolytic enzymes like ACE and MAOB fall under this category, playing roles in blood pressure regulation and dopamine metabolism, respectively. Pathway enrichment of overlapping genes depicted in Fig. 6D highlights several biological processes and biochemical pathways that are enriched in the hub genes studied. The KEGG signaling pathways depicted in Fig. 9 focuses on cocaine addiction (7 A) and dopaminergic synapse (7B). Several important hub genes involved in the cocaine addiction and dopaminergic synapse are highlighted in red. The genes including MAPK, DAT, D2R, VMAT, MAO, and NF-kB are critical hubs that mediate both the acute effects of cocaine and the long-term adaptations that lead to addiction.

### 3.6 Docking and interactions of naringenin with dopaminergic and oxido-inflammatory targets

Fig. 8 compares the molecular docking interactions of Dopamine Receptor D2 (DRD2) with naringenin and rotigotine, the reference DRD2 agonist. Naringenin had binding score of −8.3 kcal/mol with key interactions with important amino acid residues including GLU95, LEU94, LEU183, THR412, VAL91 and PRO405. Rotigotine (9.4 kcal/mol) shows a higher binding affinity compared to naringenin conducting interactions with ILE183, VAL91, LEU94, ASP114, PHE389, PHE390 and TYR416. Fig. 9 shows the docking scores and interactions between naringenin and safinamide (reference drug) with Monoamine Oxidase B (MAO-B). Both compounds have an identical docking score of −9.3 kcal/mol, indicating comparable binding affinity. Naringenin conduct non-covalent bonds with several active site residues including TYR57, TYR398, LEU171 and LEU199 while safinamide made contact points through LEU164, GLY205, GLN206, ILE316, LEU171 and LEU199. Fig. 10 shows the docking interactions of MAPK with naringenin and DA-74674. The DA-74674 has a stronger binding affinity for MAPK than naringenin, as indicated by its lower docking score. However, the score of −9.3 kcal/mol for naringenin still shows significant interaction with MAPK, suggesting that it could modulate the pathway effectively. Fig. 11 depicts the molecular docking results for the interaction between NF-κB (a transcription factor) and the two ligands (naringenin and curcumin). Naringenin had a binding energy of −5.7 kcal/mol and exhibit interactions with ASP206, HIS141, ALA242, TYR57. The lower binding energy suggests a strong interaction between naringenin and NF-κB, possibly inhibiting NF-κB activation. The interactions with amino acids such as ASP206 and HIS141 indicate that naringenin likely engages in hydrogen bonding or other polar interactions, stabilizing its binding within the active site of NF-κB. Curcumin, the reference compound had −5.5 kcal/mol interacting with key amino acid residues including TYR223, ASN244, SER208, VAL219. Curcumin also demonstrates a significant interaction with NF-κB, though with a slightly higher binding energy compared to naringenin. Curcumin forms interactions with residues like TYR223 and ASN244, which could contribute to its known anti-inflammatory effects through the inhibition of NF-κB.

**Fig. 7:**
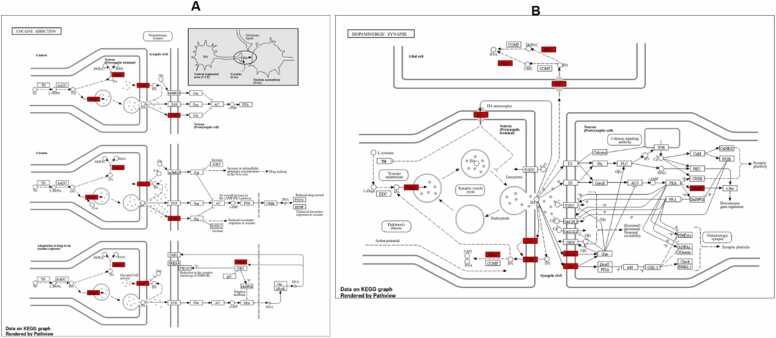
KEGG pathway mapping of naringenin-targeted genes in dopaminergic neurotransmission pathway. The figure illustrates the mapping of naringenin-targeted genes onto KEGG pathways related to cocaine addiction (A) and dopaminergic synapse (B). Red boxes highlight the genes impacted by naringenin in these pathways.

**Fig. 8:**
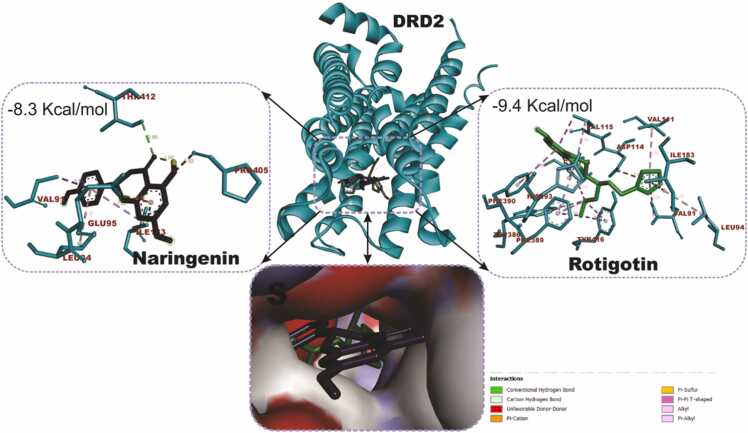
Molecular docking interactions of naringenin and rotigotin (reference drug) with the dopamine D2 receptor (DRD2). The center displays the 3D structure of DRD2 (ribbon representation), with bound ligands shown in stick representations. Naringenin binds at the active site with a binding affinity of −8.3 kcal/mol, interacting with key residues such as GLU95, VAL91, and THR412, forming hydrogen bonds and hydrophobic contacts. Rotigotin binds with a binding affinity of −9.4 kcal/mol, engaging residues like ASP114, PHE390, and ILE183. The bottom inset provides a close-up surface view of the binding pocket with the ligands docked inside.

**Fig. 9:**
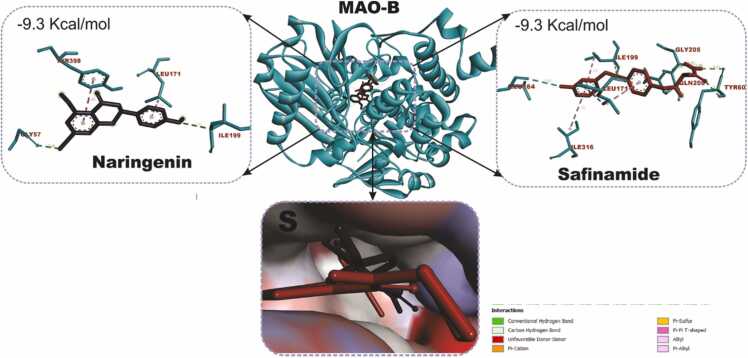
Molecular docking interactions of naringenin and safinamide (reference drug) with the monoamine oxidase B (MAO-B) enzyme. The center shows the 3D structure of MAO-B (ribbon representation), with the ligands bound in the active site. Naringenin binds to MAO-B with a binding affinity of −9.3 kcal/mol, interacting with key residues such as TYR398, LEU171, and ILE199. Safinamide also exhibits a binding affinity of −9.3 kcal/mol, engaging residues like LEU171, ILE199, and GLN206. The lower inset provides a close-up surface view of the binding pocket, showing both ligands in the active site of MAO-B.

**Fig. 10:**
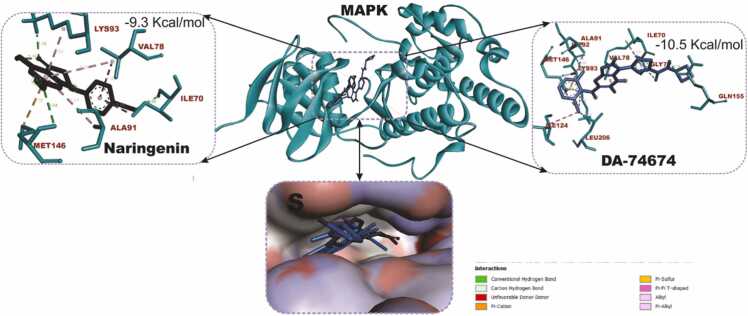
Molecular docking interactions of naringenin and DA-74674 (reference drug) with MAPK (Mitogen-Activated Protein Kinase). The central structure represents the 3D conformation of MAPK (ribbon representation) with ligands bound to the active site. Naringenin binds with a docking score of −9.3 kcal/mol, interacting with residues such as LYS93, ALA91, and MET146 through hydrogen bonds and hydrophobic contacts. DA-74674 shows a stronger binding affinity of −10.5 kcal/mol, forming interactions with key residues like LYS93, GLN155, and MET146. The lower inset provides a detailed surface view of the MAPK binding pocket, highlighting the positioning of both ligands.

**Fig. 11:**
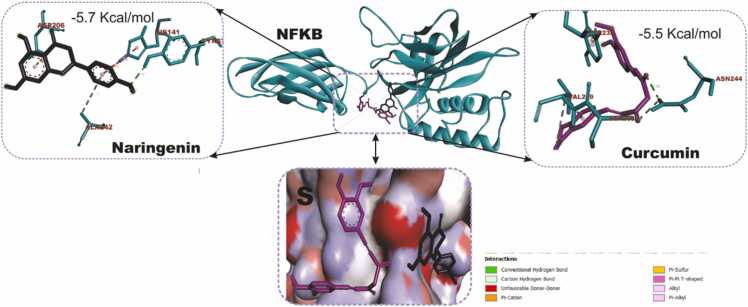
Molecular docking interactions of naringenin and curcumin (reference compound) with the NF-KB. The center shows the 3D structure of NF-KB (ribbon representation), with the ligands bound in the active site. The lower inset provides a close-up surface view of the binding pocket, showing both ligands in the active site of NF-KB.

## 4 Discussion

Our findings revealed the ameliorative role of naringenin in MPTP- induced parkinsonism using *D. melanogaster* experimental model, biological network exploration and molecular docking simulation. The beneficial effects of naringenin administration may involve several mechanisms including suppression of oxido-inflammation and modulation of dopaminergic signaling pathways in MPTP-induced PD. The hormetic response observed upon administration of naringenin alone highlights the optimal dosing range of naringenin in *D. melanogaster*. Low-to-moderate doses of naringenin (100 µM to 300 µM) seem to maintain or improve the survival of the flies. At these concentrations, naringenin’s effects may have reached an optimum to effectively reduce the levels of ROS such as NO and H_2_O_2_, and boost antioxidant defense system involving CAT and GST without overwhelming the system or producing off-target effects. Naringenin is widely reported for its potent antioxidant properties, which help in scavenging free radicals and reducing oxidative stress [59], [60], [61], a major factor in neurodegeneration caused by MPTP. Emran and Islam [61] found that lower doses of naringenin improved cognitive performance and reduced oxidative stress markers in rodent models of neurodegeneration whereas very high doses may have diminishing returns or off-target effects. Increased oxidative stress markers reported at high doses of narigenin as observed in this study suggests that, it may induce oxidative damage, which could account for the reduced survival. At these concentrations, naringenin may interact with multiple cellular pathways, leading to potential toxicity or reduced effectiveness due to off-target effects. This study also corroborates the review by Skibola and Smith [62] who noted that, excessive flavonoid supplementation can lead to imbalanced antioxidant activities and disruption of normal cellular functions.

This study demonstrates that, while MPTP treatments significantly reduced survival, motor function (negative geotaxis), and AChE activity in *D. melanogaster*, modeling neurodegenerative damage similar to PD, naringenin improves the motor function, and AChE activity, with lower doses showing the most pronounced benefits. This suggests that naringenin can mitigate the neurotoxic effects of MPTP by supporting neurotransmission (AChE) and motor function, likely through its antioxidant properties. Co-administration of MPTP and naringenin further suggests that, naringenin may lose its neuroprotective potential at higher concentrations, indicating a potential threshold beyond which it could be ineffective or even harmful. The ameliorative potential of naringenin through modulation of oxido-inflammatory pathways and dopaminergic signal pathways is widely reported in other animal models. Lou, et al. [63] reported that, administration of naringenin in mice prevented PD associated with 6-hydroxydopamine-induced nigrostriatal dopaminergic degeneration and oxidative stress through mechanism involving nuclear factor E2-related factor 2/antioxidant response element (Nrf2/ARE). Another study performed in adult Sprague–Dawley male rats also revealed that, naringenin ameliorated 6-hydroxydopamine model of PD by protecting the tyrosine hydroxylase (TH)-positive cells and increasing the level of dopamine [64]. Our study established the involvement of oxidative damage in *D. melanogaster* MPTP-induced PD model as indicated by the levels of NO, H_2_O_2_, and protein carbonyls in *D. melanogaster*. Elevated NO contributes to nitrosative stress and neuroinflammation [65], [66], [67] and is associated with protein nitration, mitochondrial dysfunction, and neuronal damage [67], [68]. By lowering NO levels, naringenin may reduce neuronal damage and inflammation as it is widely reported to modulate neuroinflammatory pathways by targeting several signal molecules such as mitogen-activated protein kinase (MAPK) [69], suppressor of cytokine signaling 3 (SOCS-3) [70], the signal transducer and activator of transcription-1 (STAT-1) [71] and Nuclear factor kappa B (NF-κB) [72]. Naringenin is known to induce a significant reduction in P65 (a subunit of NFκB) phosphorylation and nuclear translocation [71]. That is, naringenin can prevent IκB-α phosphorylation and degradation causing decreased expression and suppression of NFκB in neuroinflammation as reported by Santa-Cecília, et al. [73].

Furthermore, naringenin ameliorates the MPTP-induced decrease in key antioxidant markers such as total thiol, non-protein thiol as well as CAT and GST enzyme activities in *D. melanogaster*. This suggests that naringenin helps replenish the thiol pool and counters the oxidative damage caused by MPTP. Naringenin significantly ameliorates the loss of non-protein thiols, with higher concentrations showing greater restorative effects. This indicates that naringenin helps maintain intracellular GSH levels, which are crucial for detoxification and antioxidant defense. Earlier studies using experimental rat models, revealed that, administration of naringenin caused decreased oxidative stress markers including H_2_O_2_, protein carbonyls, 4-hydroxynonenal (4-HNE) and malondialdehyde (MDA) [74], and an increased level of glutathione (GSH), glutathione peroxidase (GPx), GR, GST, SOD, CAT and choline acetyltransferase enzyme activity [75], [76]. In addition, naringenin was reported ameliorate neurotoxicity in experimental rats by significantly reducing the activities of mitochondrial complex I–V enzymes as well as mitochondrial membrane potential [77]. Taken together, the study has demonstrated that, naringenin may exhibit its neuroprotective action through reduced neurotoxicity, inflammation and oxidative stress as well as increased antioxidant status in MPTP-induced Parkinsonism in *D. melanogaster*.

Computational analyses was performed to predict the possible biological processes, biochemical pathways, and molecular signaling involved in the ameliorative potential of naringenin. Targets network and protein-protein interactions analyses, which was focused on exploring the intricate interactions of naringenin with the MPTP inducible genes and PD targets within biological networks, revealed 54 common genes and highlights the complexity of gene interactions and shared molecular mechanisms that are crucial for both the pathophysiology of PD and the therapeutic effect of naringenin. The analysis reveals the network’s diversity of interactions (physical, co-expression, genetic interactions, etc.) indicating a multi-faceted approach to regulation and impact of naringenin. The network revealed the interconnectedness of dopaminergic signaling, neurotransmitter transport systems and oxido-inflammatory pathways as key areas influenced by naringenin, potentially offering therapeutic avenues. The hub genes identified are *DRD2/D2R, MAOB, SLC6A4, SLC6A3/DAT, DRD4/D4R, CHRNA4, DRD3/D3R, SLC18A2, CHRM2, MMP9, NFKB, CSTB, ACE, MMP2*, and *MMP1*. The topology metrics of these hub genes in the PPI network provided valuable insights into the structure and importance of these genes within the network. Few hub genes identified, particularly *DRD2, MAOB, SLC6A4*, and *MMP9*, exhibit significant centrality and connectivity within the network, indicating their critical roles in the mechanism. These genes are not only highly interconnected but also strategically positioned to control the flow and stability of information within the network, making them potential key targets for therapeutic strategies in Parkinsonism. The *DRD2* which was highlighted as a major hub, plays a critical role in dopaminergic signaling and is crucial in PD pathology [78]. The *DRD4* similar to *DRD2*, is involved in dopaminergic signaling. The *MAOB*, another significant hub gene, is involved in the breakdown of dopamine and is often implicated in PD [79]. The *SLC6A3* (Dopamine Transporter, DAT) gene encodes the dopamine transporter and is crucial in dopamine reuptake and clearance from synaptic spaces. Its regulation is essential for maintaining dopamine levels, making it a critical player in Parkinsonism. Other Hub Genes including *SLC6A2* and *SLC6A*4 are part of the solute carrier family, involved in neurotransmitter transport [80], indicating that naringenin might influence a broad range of neurotransmitter systems beyond dopamine.

Enrichment of the hub genes in the various biological processes suggests that the hub genes are central to neuroprotective mechanisms, particularly against oxidative stress, chemical toxins, and dysregulated transport systems. Damaged mitochondria produce ROS, which leads to oxidative stress, causing damage to cellular structures like proteins, lipids, and DNA [81]. This mitochondrial impairment, along with defective mitophagy (the process of removing damaged mitochondria), contributes to neuronal degeneration [82]. In PD, various environmental chemical toxins contribute to dopaminergic neuron death through induction of oxidative stress, making this biological process crucial in understanding the disease mechanism [83], [84]. In addition, high enrichment in regulation of transport suggests the involvement of hub genes in regulating the transport of molecules across cell membranes. Dysregulated transport of dopamine and other neurotransmitters is a hallmark of PD, particularly involving the SLC6 family of transporters including SLC6A3, SLC6A4, which are key regulators of neurotransmitter homeostasis [85], [86]. The hub genes collectively underscore the complex interplay between dopaminergic, serotonergic, cholinergic, antioxidant and inflammatory pathways in MPTP-induced toxicity and naringenin-induced neuroprotection. This further supports the widely reported antioxidant potential of naringenin in PD and other neurodegenerative disorders [87], [88], [89].

Cellular component enrichment of the hub genes indicate that they are primarily localized and most active in synaptic regions, the plasma membrane, and associated vesicular components. This suggests their critical roles in synaptic transmission, neurotransmitter regulation, and neuronal communication, all of which are essential for proper dopaminergic function. Enrichment in the synaptic regions and membranes is particularly significant in the context of PD, where synaptic dysfunction, particularly in dopaminergic neurons, is a major pathophysiological factor. Genes like *SLC6A3* and SLC6A4 (dopamine and serotonin transporters, respectively) are critical to synaptic function [90]. The plasma membrane, especially in neurons, is key to maintaining ion gradients and facilitating neurotransmitter release and uptake. Genes involved in neurotransmitter transport (*SLC6A3, SLC6A4*) and receptors (DRD2, DRD3) are central to this function. Dopamine receptors (*DRD2, DRD4*) and transporters (*SLC6A3, SLC18A2*) are integral components of the membrane, regulating dopamine dynamics in neurons. Genes like *SLC18A2*, the gene that encodes vesicular monoamine transporter 2 (VMAT2) are involved in packing neurotransmitters into vesicles, essential for synaptic transmission [91]. Disruption of vesicular trafficking is implicated in PD pathology, especially in dopamine release [92]. Naringenin is known to enhance synaptic plasticity, the ability of neurons to strengthen or weaken their connections based on activity [87], [93], [94]. This is vital for learning and memory, and naringenin’s positive impact on synaptic plasticity could explain its cognitive-enhancing effects in neurodegenerative diseases and brain injury models. Taken together, the enrichment in synaptic components and vesicles points to their involvement in processes like neurotransmitter release, synaptic plasticity, and potentially in the pathogenesis of neurodegenerative disorders like PD.

Molecular function enrichment of the hub genes revealed that they are primarily involved in neurotransmitter receptor activity, enzyme regulation (including metalloproteinases and neurotransmitter-degrading enzymes), and binding functions related to cellular signaling. These functions are crucial in the context of PD, where neurotransmitter imbalance, oxidative stress, and neuroinflammation are prominent [95], [96]. The postsynaptic neurotransmitter receptor activity involves the function of proteins that act as receptors for neurotransmitters in the postsynaptic membrane, particularly in neurotransmission. Genes linked to dopamine (e.g., *DRD2, DRD3, DRD4*) and acetylcholine receptors (e.g., *CHRNA4, CHRM2*) are key players here, as they are vital for proper synaptic signaling. Neurotransmitter Receptor Activity is a broader category that include the activity of receptors that bind neurotransmitters such as dopamine, serotonin, and acetylcholine, relevant to genes like *SLC6A3* (dopamine transporter), *SLC6A4* (serotonin transporter), and *CHRM2* (muscarinic receptor). The pathway enrichment analysis revealed the top enriched pathways include those related to dopaminergic neurotransmission (cocaine addiction, dopaminergic synapse), inflammatory signaling (IL-17, T cell receptor signaling), and metabolic processes (cytochrome P450, fluid shear stress, atherosclerosis). Cocaine Addiction pathway map generally involves neurotransmitter signaling, particularly dopamine. Hub genes including *DRD2, DRD3*, and *SLC6A3* (dopamine transporter) are likely involved here. In PD, dopamine dysregulation plays a significant role, and this pathway suggests that addiction-related dopamine signaling may overlap with mechanisms affected in PD. The Cytochrome P450 enzymes are involved in the metabolism of various drugs, and genes like *MAOB* and SLC6A4 (serotonin transporter) could play roles in drug metabolism and neurotransmitter processing. This pathway indicates that altered drug metabolism indicated by cytochrome P450 activity might be significant in MPTP-induced parkinsonism which can be might modulated by naringenin, improving drug metabolism and reducing neurotoxicity. Inflammatory responses driven by IL-17 Signaling Pathway are implicated in the pathway analysis. Neuroinflammation plays a key role in PD progression. Microglia, the brain's resident immune cells, become activated in response to neuronal injury or the presence of alpha-synuclein aggregates. While microglial activation is initially protective, chronic activation leads to the release of pro-inflammatory cytokines (e.g., TNF-α, IL-1β) and ROS, exacerbating neuronal damage [97], [98]. Emerging evidence suggests that systemic immune system dysregulation may also contribute to PD pathogenesis, as increased levels of peripheral inflammatory markers have been detected in patients with PD[99]. This could be linked to the hub NFKB which plays a central role in inflammatory responses. In addition, T cell signaling pathway plays a role in the immune response is implicated which may involve genes like *NFKB* and *MMP*s central to immune and inflammatory responses. Naringenin may modulate T cell responses, reducing neuroinflammation and potentially protecting against disease progression. Microglia are the immune cells of the central nervous system and become activated during neuroinflammation, which is a hallmark of neurodegenerative diseases. Naringenin is known to exhibit anti-inflammatory potential by inhibiting microglial activation [100]. It can inhibit overactivation of microglia and astrocytes, thereby reducing the production of pro-inflammatory cytokines (such as TNF-α, IL-1β, and IL-6) and nitric oxide (NO) [94]. This helps to prevent chronic neuroinflammation and its associated neuronal damage. Naringenin is also known to inhibit the NF-κB (nuclear factor kappa B) signaling pathway, which is a key regulator of inflammation [101]. By blocking the nuclear translocation of NF-κB, naringenin reduces the expression of pro-inflammatory genes, thereby attenuating inflammation in the brain.

Dopaminergic Synapse pathway is critical in PD, as it involves dopamine synthesis, release, and signaling. Genes such as *DRD2, DRD3, DRD4, SLC6A3*, and *MAOB* are involved in dopaminergic synapse function. Naringenin’s effect on this pathway could help restore dopaminergic signaling, potentially improving motor symptoms associated with Parkinsonism. Also, neuroactive ligand-receptor interaction pathway including interactions between neurotransmitters and their receptors is implicated in this study. Genes involved in dopamine, serotonin, and acetylcholine signaling are likely enriched in this category, relevant to DRD2, SLC6A3, SLC6A4, and CHRNA4. In general, naringenin’s neuroprotective role may involve restoring dopamine balance, reducing neuroinflammation, and improving vascular and metabolic health. The pathways highlighted suggest overlapping mechanisms between Parkinsonism pathology and other diseases or conditions, underscoring the complexity of neurodegeneration and the potential multi-target effects of naringenin. Visualization of the dopaminergic neurotransmission pathway map viz: cocaine addiction and dopaminergic synapse revealed the central role of the hub genes such as *DAT, MAPK, DRD2/D2R, VMAT, MAO* and *NF-kB*. The KEGG dopaminergic pathway illustrates the complex regulation of dopaminergic signaling and how various proteins various genes are involved in the modulation of the synthesis, release, action, and degradation of dopamine.

While network pharmacology provides a system-wide perspective of drug-target interactions by examining the interconnected nature of biological pathways and networks, molecular docking offers detailed insights into the specific interactions between drugs and their targets at the atomic level. To clarify such interaction, important hub genes that show centrality and connectivity within the gene network and that are suggested to play important role in the dopaminergic neuroprotection were docked with naringenin. The DRD2 and MAOB corresponding to dopaminergic targets as well as MAPK and NFKB in the oxido-inflammatory process are not only suggested to be strategically positioned to control the flow and stability of information within the network, they are involved various biological, cellular and molecular processes that may underlie neuroprotective potential of narigenin making them potential key targets for therapeutic strategies in PD. In this study, 4 targets were docked with naringenin in comparison with their respective cocrystalized/reference drugs. DRD2 exhibits a moderately strong binding affinity for naringenin, suggesting that naringenin may effectively bind and potentially modulate the receptor in a biological context. The higher binding affinity of rotigotine compared to naringenin is expected since rotigotine is an established dopaminergic agonist used in the treatment of PD. The more negative docking score indicates stronger binding to the receptor, making it a more potent activator of DRD2. Both compounds engage in similar hydrophobic interactions with residues like VAL91, LEU94, and ILE183. However, rotigotine benefits from additional interactions, such as π-π stacking with PHE389/390 and hydrogen bonding with ASP114 and TYR416, making its overall binding more stable. The binding affinity of naringenin suggests it could contribute to dopaminergic modulation, supporting its potential neuroprotective effects, especially in complementary or alternative treatment strategies targeting PD. Docking MAO-B with the ligands shows that, naringenin had identical binding affinity as compared to safinamide suggesting that it could be as effective as safinamide in inhibiting MAO-B. This implies that Naringenin has strong potential as an alternative or adjunct therapy to traditional MAO-B inhibitors like Safinamide. The two compounds share some common MAO-B interactions, particularly with residues LEU171 and LEU199, which help stabilize both compounds in the hydrophobic binding pocket of MAO-B. While Naringenin engages with TYR57 and TYR398, Safinamide forms additional interactions with GLY205, GLN206, and ILE316, suggesting slightly more diverse contact points, which could translate to nuanced differences in their inhibitory mechanisms. Both compounds exhibit strong binding to MAO-B, supporting their role in the inhibition of dopamine degradation. Naringenin shows promise as a natural compound with similar efficacy to Safinamide, a clinically used drug. As described earlier, MPTP is metabolised to MPP+ by the monoamine-oxidase B (MAO-B), which is then selectively taken up by the dopaminergic neurons, thus causing their death [2]. In the brain, MPTP. Inhibiting MAO-B would impede MPTP uptake as well as contribute to higher dopamine levels, which is crucial for alleviating the motor symptoms of PD. Given the strong binding of naringenin and its natural origin, it could be a valuable candidate for further investigation in PD therapy, potentially complementing or offering an alternative to traditional MAO-B inhibitors.

Naringenin's interaction with key residues (such as Lys93 and Met146) of the active site of MAPK and the relatively strong docking score of −9.3 kcal/mol suggest that it could effectively modulate MAPK signaling. This interaction may contribute to its potential therapeutic effects in MPTP-induced PD, offering neuroprotection and reducing neuronal damage. Naringeni interacts with Lys93, Val78, Ile70, Ala91, and Met146 in MAPK through multiple hydrogen bonds and hydrophobic interactions. The Lys93 and Ala91 likely provide stabilizing hydrogen bonds. Hydrophobic interactions with Val78, Ile70, and Met146 anchor Naringenin in the binding site. This suggests that Naringenin has a stable binding within the MAPK active site, which might contribute to its potential inhibitory effect on MAPK signaling. In PD, inhibition of MAPK pathways has been linked to reduced neuroinflammation and protection against neuronal death, which is crucial in mitigating disease progression. MAPK signaling is involved in regulating cell responses to oxidative stress and apoptosis, both of which are important in the pathology of PD. Naringenin's ability to bind MAPK could contribute to its neuroprotective effects by modulating this pathway, potentially reducing neuroinflammation and protecting dopaminergic neurons from degeneration. Both naringenin and curcumin may exert neuroprotective effects by modulating the NF-κB pathway, which is implicated in neuroinflammation, and the progression of PD. Naringenin's slightly more favorable binding energy suggests it could be an effective modulator of NF-κB, potentially reducing inflammation and oxidative stress in MPTP-induced PD models. Thus, the docking results highlight naringenin as a promising candidate for attenuating NF-κB activity and provide a mechanistic rationale for its ameliorative role in neuroinflammatory conditions like PD.

## 5 Conclusion

This study focused on probing the neuroprotective potential of naringenin in an MPTP-induced *D. melanogaster* model of PD, integrating experimental findings with computational insights. Our results demonstrate that naringenin exhibits hormetic behavior, where low-to-moderate doses confer significant neuroprotection. These effects are mediated through antioxidant, anti-inflammatory, and dopaminergic pathways, with optimal dosing playing a crucial role in efficacy. Network pharmacology and molecular docking analyses support the interactions of naringenin with key molecular targets, further supporting its potential as a therapeutic candidate for PD. Despite these promising findings, further research is necessary to enhance their translational relevance. Rigorous clinical trials are needed to validate naringenin’s neuroprotective effects in PD patients and establish its optimal dosing in humans. Also, experimental validation of its interactions with key molecular targets identified through computational studies will provide deeper mechanistic insights. Additionally, investigating the long-term effects of naringenin supplementation and its precise dose-response relationship will be essential for defining its therapeutic window and potential clinical applications.

## CRediT authorship contribution statement

**Ogunyemi Oludare Michael:** Writing – review & editing, Writing – original draft, Visualization, Software, Methodology, Investigation, Formal analysis. **Okonta Clive:** Writing – review & editing, Writing – original draft, Methodology, Investigation, Formal analysis, Data curation. **Abolaji Amos Olalekan:** Writing – review & editing, Validation, Supervision, Resources, Project administration, Investigation, Conceptualization. **Olabuntu Babatunde:** Visualization, Software, Methodology, Formal analysis, Data curation.

## Declaration of Competing Interest

The authors declare that they have no known competing financial interests or personal relationships that could have appeared to influence the work reported in this paper.

## Data Availability

No data was used for the research described in the article.
